# Mindfulness-Based Cognitive Therapy in Recurrent MDD Patients With Residual Symptoms: Alterations in Resting-State Theta Oscillation Dynamics Associated With Changes in Depression and Rumination

**DOI:** 10.3389/fpsyt.2022.818298

**Published:** 2022-03-07

**Authors:** Jing Wang, Feng Ren, Bingling Gao, Xin Yu

**Affiliations:** ^1^National Health Commission Key Laboratory of Mental Health (Peking University), National Clinical Research Center for Mental Disorders, Peking University Sixth Hospital (Institute of Mental Health), Beijing, China; ^2^Peking University Shougang Hospital, Beijing, China

**Keywords:** major depressive disorders, residual symptoms, rumination, electroencephalography, theta rhythm, mindfulness-based cognitive therapy

## Abstract

Many patients with major depressive disorder (MDD) suffer from residual symptoms. Rumination is a specific known risk factor for the onset, severity, prolongation, and relapse of MDD. This study aimed to examine the efficacy and EEG substrates of mindfulness-based cognitive therapy (MBCT) in alleviating depression and rumination in an MDD population with residual symptoms. We recruited 26 recurrent MDD individuals who had residual symptoms with their current antidepressants to participate in the 8-week MBCT intervention. We evaluated the efficacy and changes in the dynamics of resting-state theta rhythm after the intervention, as well as the associations between theta alterations and improvements in depression and rumination. The participants showed reduced depression, enhanced adaptive reflective rumination, and increased theta power and phase synchronization after MBCT. The increased theta-band phase synchronizations between the right occipital regions and the right prefrontal, central, and parietal regions were associated with reduced depression, while the increase in theta power in the left parietal region was associated with improvements in reflective rumination. MBCT could alleviate depression and enhance adaptive, reflective rumination in recurrent MDD individuals with residual symptoms through the modulation of theta dynamics in specific brain regions.

## Introduction

Major depressive disorder (MDD), one of the most common psychiatric disorders with high prevalence, heterogeneous causes, and variable treatment response, is associated with the number of years lived with disability at a global level (1). Many patients, even those with marked improvements after first-line treatment, suffer from depression-related symptoms (called residual symptoms) of various intensities and have a higher probability of experiencing a chronic course and relapse of depressive episodes (2, 3).

Rumination, characterized as repetitive, prolonged, self-reflective, and uncontrollable focus on symptoms of distress and its potential antecedents or repercussions (4), has been well-accepted to directly contribute to more severe depressive symptoms, chronicity of the depressive episode, and a higher risk of depressive relapse (4–6). Given its robust relationship with depression, rumination has been identified as a malleable target for the prevention of and intervention for MDD (7).

Mindfulness-based cognitive therapy (MBCT) is an evidence-based, manualized and systematic 8-week group training that combines traditional elements of cognitive-behavior therapy with novel mindfulness meditation techniques in an integrated manner (8). MBCT trains participants to recognize and disengage from maladaptive automatic cognitive patterns and to develop a non-judgemental and compassionate attitude toward their own cognitions and feelings (9). MBCT has been demonstrated to be effective in reducing relapse (10) and depressive symptoms in currently depressed patients (11). Recently, MBCT effects on rumination during depressive episodes was also reported (9, 12, 13). However, it is not clear whether MBCT is effective in alleviating depression and rumination in patients who have residual symptoms with their current antidepressants. It is worth investigating the efficacy of MBCT and exploring the underlying neurophysiological substrates.

Electroencephalography (EEG) studies have identified some changes in brain oscillations as a mechanism of mindfulness, such as alpha power, theta power, and synchronization (14–16). It is accepted that theta band subserves recruitment of meta-awareness and cognitive control to sustain the meditative state (17), while alpha band maintains the cortical arousal to avoid perturbations by external stimuli (18). Evidence from functional magnetic resonance imaging (fMRI) studies have suggested that the default mode network (DMN), frontoparietal network (FPN), cingulo-opercular network (CON), and the reorganization of internetworks are also important in mindfulness-based interventions (19–22). However, the mechanisms by which mindfulness-based neuroplasticity alleviates depression and rumination require further investigation. In search of the neural substrates of depression and rumination, EEG and fMRI studies have demonstrated altered structures in dynamic EEG oscillatory patterns and aberrant brain functional networks in MDD (23–25). Evidence from fMRI has identified that rumination is related to aberrant activity and connectivity of various regions and networks, such as key nodes in the DMN, including the inferior parietal cortex (IPC), posterior cingulate cortex (PCC), precuneus, and so on (26–29), but thus far, there is very little information on the effects of rumination on power and phase coupling in different frequency ranges.

Considering the evidence cited above and the role of theta rhythms in emotion, cognition and meditation, we speculated that MBCT would moderate the aberrant dynamics in theta oscillations in specific brain regions in the resting state without external stimuli to improve depression and rumination in MDD patients who have residual symptoms with their current antidepressants. In the current study, we aimed to achieve three objectives: (a) investigate the efficacy of MBCT in alleviating depression and rumination in a recurrent MDD population with residual symptoms with their current antidepressants, (b) identify the changes in the dynamics in theta rhythm (including power and interregional phase synchrony) affected by an 8-week MBCT, and (c) explore the associations between MBCT-induced theta alterations and score changes in depression and rumination (assessed by questionnaire).

## Materials and Methods

### Participants

A total of 45 recurrent MDD patients with residual symptoms (age range, 18–65 years) were recruited between March 1, 2017, and September 1, 2017, from outpatient psychiatric units at Peking University Shougang Hospital. All participants met the criteria for a diagnosis of recurrent MDD based on the International Statistical Classification of Diseases and Related Health Problems, Tenth Revision (ICD-10), which includes a score of more than 7 and less than 18 on the 17-item Hamilton Rating Scale for Depression (30) (HDRS_17_). At the time of baseline assessment, all patients had been taking standardized antidepressant treatment, without adjustments in medicine type and dosage for more than 8 weeks. The exclusion criteria were (a) psychotic episodes, dementia, bipolar disorder, substance dependence or abuse; (b) any unstable medical conditions or known neurological disease; (c) pregnancy or a plan to become pregnant during the study; and (d) any experience with mindfulness meditation, regular yoga or psychotherapy prior to this study.

The drop-out criteria were as follows: (a) symptom fluctuation or aggravation that caused the patient to be unable to continue to participate or mania symptoms emerged during the study; (b) less than 4 MBCT sessions completed; and (c) changes in antidepressants or increases in medicine dosages during the study.

Finally, 35 participants completed the 8-week intervention, and EEG data were not collected from 9 of them. Therefore, complete datasets were available from 26 participants (mean age = 49.46 ± 13.11 years; 7 males) for data analysis in this study. The study was approved by the Ethics Committee of the Peking University Shougang Hospital. Participants gave their written informed consent prior to participating in the study and were treated in accordance with the Declaration of Helsinki.

### Questionnaire Assessment

Prior to receiving an 8-week MBCT training course and after 4 weeks and 8 weeks of training, the participants completed the questionnaire battery, which included (a) the HDRS_17_, the rating scale for depression, administered by certified psychiatrists who did not participate in the MBCT intervention—remission of MDD was defined as an HDRS_17_ total score ≤ 7 at week 8; (b) the 21-item Beck Depression Inventory (BDI), the self-evaluated scale for depression; (c) the well-validated Chinese version of the 22-item Ruminative Response Scale (RRS) (31), with the two facets (32) of brooding rumination (RRS-B, 5 items), which is a maladaptive process, and reflective rumination (RRS-R, 5 items), which is an adaptive strategy because it may facilitate problem-solving, were calculated; and (d) the Chinese version of Five Facet Mindfulness Questionnaire (FFMQ), one of the well-studied and reliable measures of mindfulness.

### Mindfulness-Based Cognitive Therapy

The adopted intervention was standardized antidepressants combined with the 8-week group-based MBCT program (33), which consisted of one group meeting per week (2.5 h each time) and recommended daily home practice (30–40 min per day) for 8 weeks. The courses were designed based on “Mindfulness-based cognitive therapy for depression” (33). The first 4 weeks of the course led the participants to practice basic skills of mindfulness, recognize the automatic ways of acting, and direct their attention to the present moment by focusing on the breath and body sensations through mindfulness practices. The 5th to 8th weeks of the course aimed to sharpen participants' mindfulness practice to develop the participants' skills in handling depressed mood and aversive cognition. All procedures were guided by a group therapist certificated to facilitate mindfulness interventions. The participants were led by the therapist with guided meditation training and in-class practices, experiential exercises, and discussions of their daily practices. During the whole MBCT intervention, the participants needed to keep their antidepressant treatment unchanged.

### EEG Acquisition and Preprocessing

Prior to receiving an 8-week MBCT training course and after the 8-week training, resting-state eyes-closed EEG signals were continuously collected for 10 min using the EEG data acquisition system (ANDETE-2010, Guangzhou Million Andey Electronic Technology Co., Ltd.) with a standard 18-channel cap based on the International 10/20 extended system of electrode positions. The EEG signals were amplified with bandpass filtering at 0.5–200 Hz and a sampling rate of 512 Hz, referenced to the bilateral mastoids. All subjects remained lying in a relaxed and comfortable position in a sound attenuating room, remained awake with their eyes closed, and did not to move or talk.

Signals were analyzed offline with the MATLAB R2014a (MathWorks, Natick, MA, USA)-based EEGLAB toolbox (http://sccn.ucsd.edu/eeglab/). EEG data were rereferenced to average and bandpass filtered in the range of 0.5–45 Hz to avoid the interference of 50-Hz signals. Conspicuous baseline drift and artifacts caused by eye movement, significant muscle activity and movement were eliminated by visual inspection of the time series.

### EEG Signal Processing

#### Power Spectrum Analysis

To obtain the power of the resting-state EEG data, the wavelet-based power spectrum analysis method with a wavelet central angle frequency of 6 (ω = 6) (34, 35) was employed. Briefly, the signals were divided into a series of 2-s epochs. Power values of each epoch across all electrodes were calculated from 0.5 to 45 Hz, with a step size of 0.5 Hz. Finally, the power values of the whole signal were obtained by averaging the power over all epochs. The following five frequency bands were examined: 1–4, 4–8, 8–13, 13–30, and 30–45 Hz, corresponding to the delta, theta, alpha, beta and gamma bands, respectively.

#### Theta-Band Phase Locking Value (PLV)

Interregional phase synchrony in the theta band was quantified by calculating the theta-band phase locking value (PLV) (36). Briefly, EEG signals from single 2-s-epochs were first transformed into narrowband signals in the theta band through bandpass filtering (4–8 Hz). The instantaneous phase for each time point was calculated from the narrowband signal by Hilbert transform. The PLV between two electrodes was calculated for each time point by averaging the phase difference across all epochs of the whole signal.

### Statistical Analyses

First, intervention effects were evaluated using paired *t*-tests on the clinical, psychological and the two EEG variables (powers and pairwise PLV), with effect sizes quantified using Cohen's *d*. Second, we performed linear regression analysis to examine the relationships between the changes in those significant EEG variables and those in the HDRS and RRS scores (reflective rumination). Considering the multiple comparisons, the significance level of *P* < 0.05 was corrected by a false discovery rate (FDR).

## Results

### Intervention Effects on Clinical and Psychological Characteristics

Table 1 shows the summary of the clinical and psychological variables for the recurrent MDD individuals with residual symptoms. The overall remission rate was 88.46% at week 8 (only 3 participants had HDRS scores > 7). Compared with the pre-MBCT scores, HDRS scores and BDI scores significantly decreased after MBCT, whereas the mindfulness level (FFMQ score) significantly increased. Although RRS total scores did not significantly decrease, the percentage of reflective facets (RRS-R) was markedly enhanced.

**Table 1:** Changes in clinical and psychological variables.

	**Pre-MBCT**	**Post-MBCT**	***P*** **-value**	**Cohen's D**
	**(Mean ±SD)**	**(Mean ±SD)**		
HDRS17 score	14.54 ± 3.625	4.31 ± 2.739	<0.0001^***^	2.119
Remission rate	NA	88.46% (23/26)	NA	NA
BDI score	9.92 ± 5.564	6.62 ± 4.759	0.0142^*^	0.516
FFMQ score	90.58 ± 13.615	117.38 ± 16.613	<0.0001^***^	1.723
RRS total score	38.73 ± 9.644	38.65 ± 9.234	0.9723	0.007
RRS-B (%)	22.71 ± 3.314	22.84 ± 2.772	0.8789	0.029
RRS-R (%)	22.40 ± 4.491	25.07 ± 5.396	0.0307^*^	0.4489

*MBCT, mindfulness-based cognitive therapy; HDRS17, the 17-item Hamilton Depression Rating Scale; BDI, Beck Depression Inventory; FFMQ, Five Facet Mindfulness Questionnaire; RRS, Ruminative Response Scale; RRS-B, brooding rumination; RRS-R, reflective rumination*.

* *P < 0.05*,

*** *P < 0.001, NA, not applicable*.

### Alterations in EEG Theta Activity After MBCT

**Figure 1:**
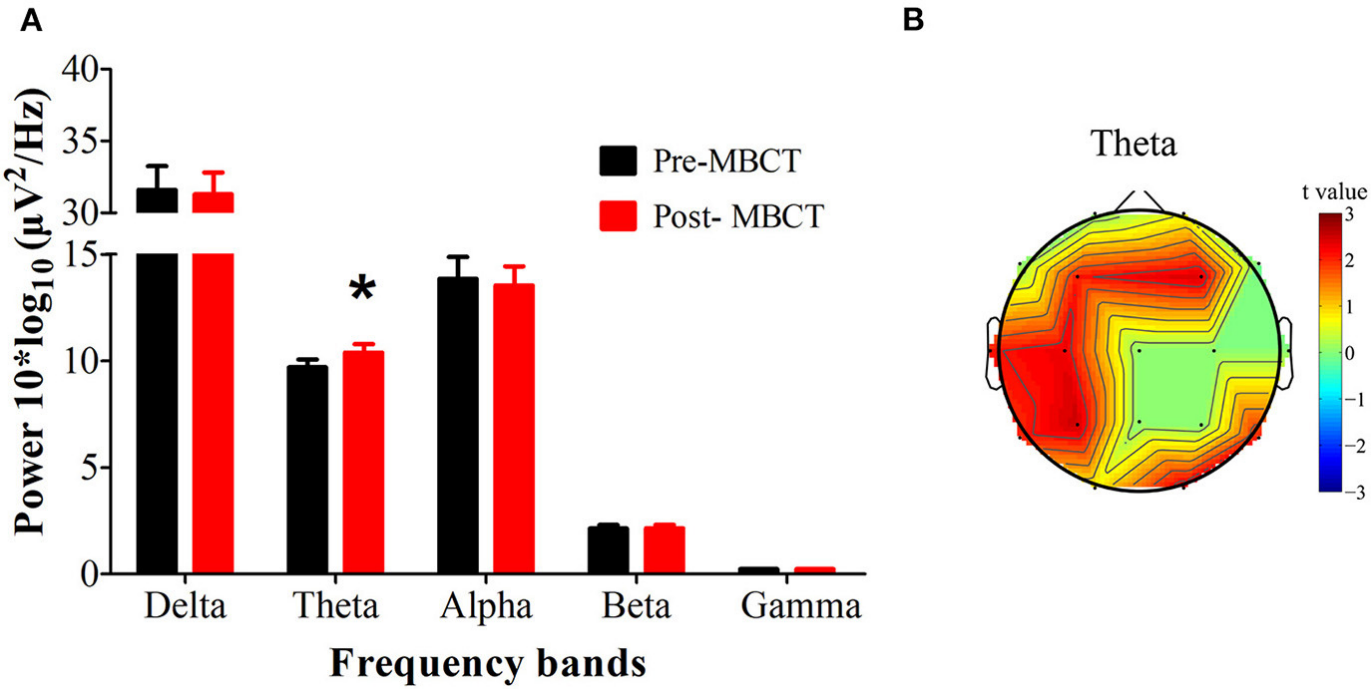
Changes in the EEG power spectra after the 8-week MBCT intervention. **(A)** The average absolute EEG power in 5 frequency bands before (black columns) and after MBCT (red columns). The Y-axis represents power values, and the X-axis represents the frequency bands. A marked increase in power in the theta frequency was observed after intervention. ^*^P < 0.05, FDR uncorrected. All data are expressed as the means ± SEM (*n* = 26). **(B)** Topographic distribution of statistically significant theta power after MBCT compared to baseline before MBCT. Values are color coded: red = *P* < 0.05; green = not significant; uncorrected for the number of electrodes tested.

**Figure 2:**
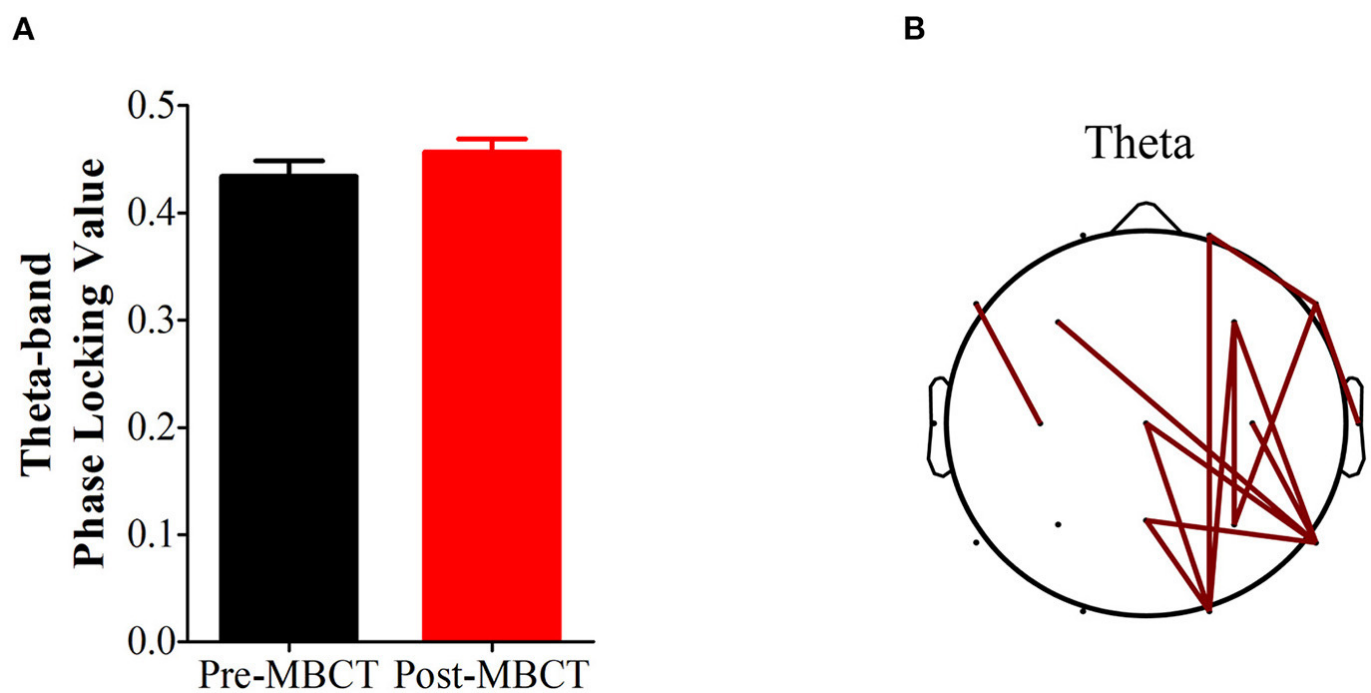
Changes in theta-band phase synchronization after the 8-week MBCT intervention. **(A)** Bar plot of theta-band phase synchronization after MBCT compared with that before MBCT; *P* = 0.0755. Bars and vertical lines indicate mean and standard errors, respectively. **(B)** Topological comparison of theta-band phase locking value after MBCT intervention. Each edge represents a significantly reduced theta-band phase locking value after the intervention based on a *P*-value of 0.05 (FDR corrected).

Figures 1, 2 present the significant changes in resting-state theta activities in the recurrent MDD population with residual symptoms.

After the 8-week MBCT intervention, we observed increased theta power (*P* = 0.0357, *d* = 0.433), especially in the bilateral frontal lobes (electrode F3, F4), the left tempo-parietal region (electrode C3, P3, T3) and the right occipital region (electrode O2) (*P* < 0.05, FDR uncorrected; Figure 1).

There was a marginally significant increase in theta-band phase synchronization (*P* = 0.0755) after the 8-week MBCT, and we further observed that the markedly increased theta-band phase synchronizations were mainly located in the right hemisphere (*P* < 0.05, FDR corrected; Figure 2).

### Depression-Related, Reflective Rumination-Related Theta Changes After MBCT

**Figure 3:**
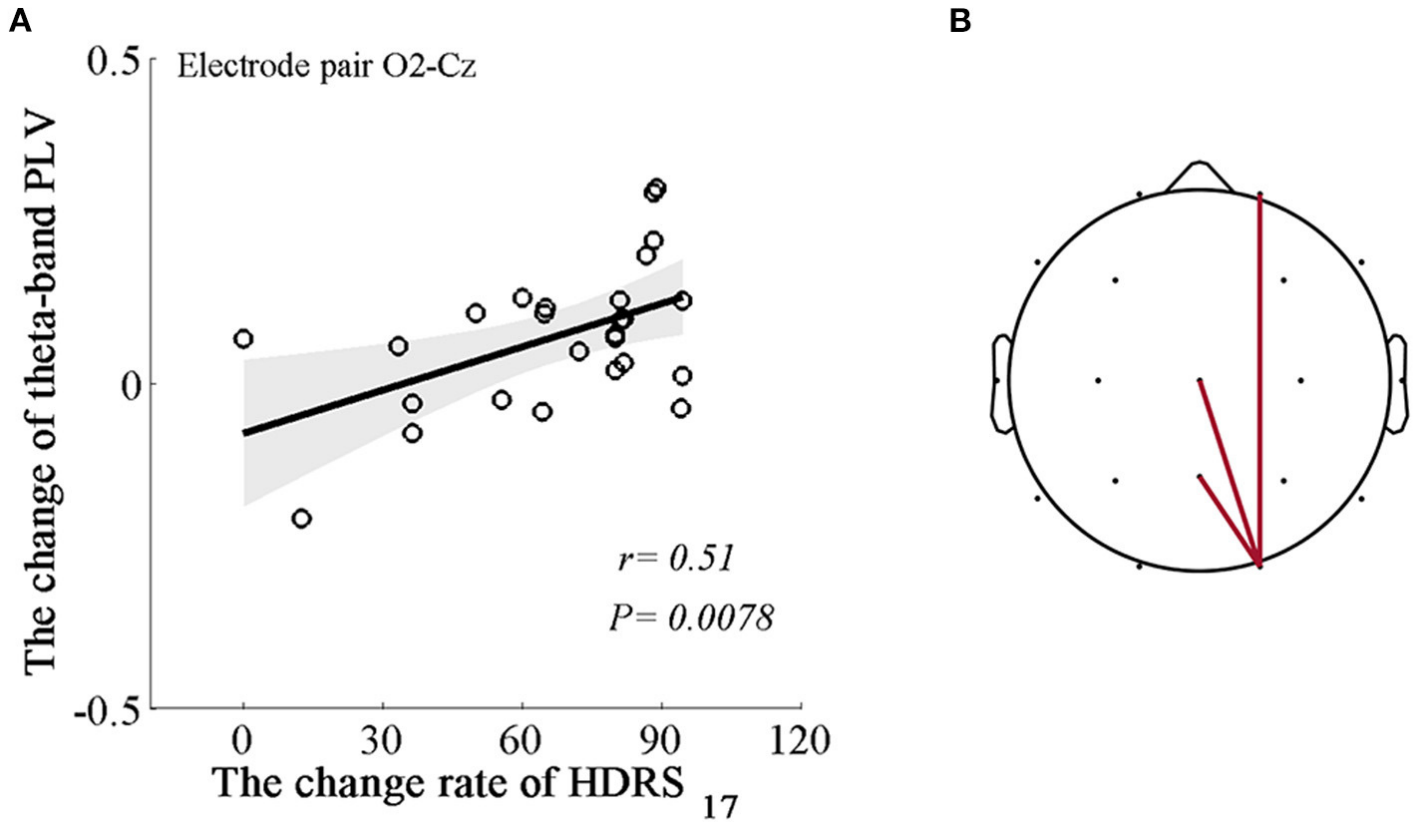
Associations between the changes in the dynamics of the EEG theta rhythm and the change rate of HDRS scores. **(A)** An example of the positive correlation between Δtheta PLV at the right occipital-central areas (electrode pair O2-Cz) and the change rate of HDRS scores (*r* = 0.51, *P* = 0.0078). Shaded areas delineate the confidence interval of 95%. **(B)** Topological correlations between Δtheta PLV and the change rate of HDRS scores. Each edge represents significantly positive correlations based on a *P*-value of 0.05.

We applied regression analysis to the significant theta variables and illustrated prominent associations between power changes in the theta bands (Δtheta power) and the change rate of HDRS scores and between the PLV changes in the theta band (Δtheta PLV) and the change rate of HDRS scores after MBCT. We noticed that the Δtheta PLV between the right occipital region and the right prefrontal, central, and parietal regions (electrode pairs: O2-Fp2, O2-Cz, O2-Pz, r_s_ = 0.39–0.51; *P*_s_ = 0.0078–0.048, FDR uncorrected), not Δtheta power, were positively associated with the change rate of HDRS scores (Figure 3).

**Figure 4:**
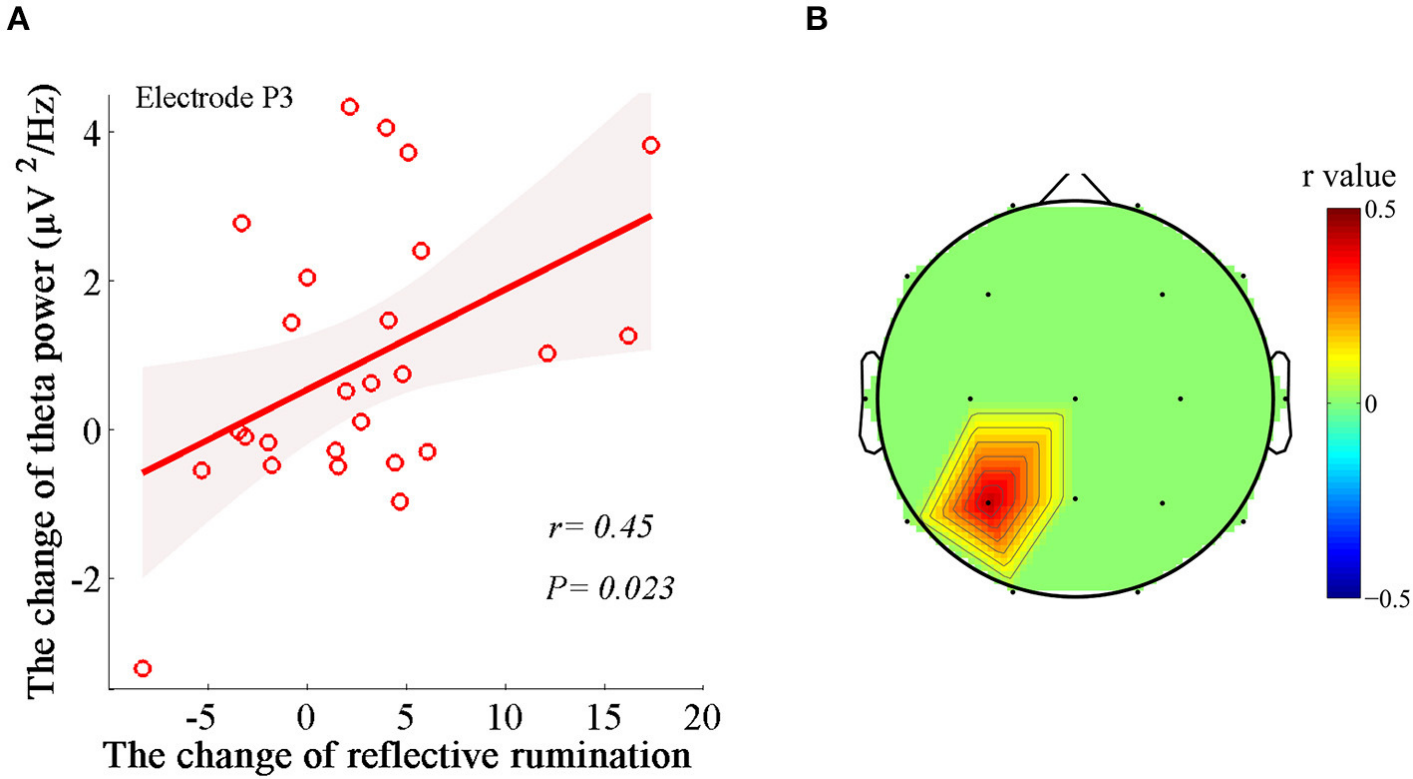
Associations between the changes in the dynamics of EEG theta rhythm and the changes in reflective rumination. **(A)** Positive correlation between Δtheta power in the left parietal region (electrode P3) and ΔRRS-R (*r* = 0.45, *P* = 0.023). Shaded areas delineate the confidence interval of 95%. **(B)** Topological correlations between Δtheta PLV and ΔRRS-R.

Similarly, we applied regression analysis to the significant theta variables and illustrated the prominent associations between the power changes in the theta bands (Δtheta power) and the changes in RRS-R percentages (ΔRRS-R) and between the PLV changes in the theta band (Δtheta PLV) and the changes in RRS-R percentages (ΔRRS-R) after MBCT. The ΔRRS-R was significantly associated with only Δtheta power in the left parietal region (electrode P3; *r* = 0.45, *P* = 0.023; Figure 4). No significant correlations were found between Δtheta PLV and ΔRRS-R.

## Discussion

The efficacy and changes in resting-state EEG activities after 8 weeks of MBCT intervention were evaluated among recurrent MDD participants who had residual symptoms with their current antidepressants. We evaluated the MBCT intervention effect by observing changes in the questionnaire indices relative to their pretraining scores. The results indicated significant reductions in depressive symptoms and increases in mindfulness level and reflective rumination, denoting the alleviation of abnormal rumination among the depressed individuals with residual symptoms after MBCT. With regard to EEG dynamics, the results indicated that MBCT increased resting-state theta power across widely distributed regions and phase synchronization mainly in the right hemisphere. Furthermore, the change rate of HDRS scores showed positive correlations with theta-band phase synchronizations between the right occipital regions and the right prefrontal, central, and parietal regions, while the RRS-R exhibited a positive correlation with theta power in the left parietal region, implying that specific theta activities participated in the alleviation of depressive symptoms and regulation of ruminative responses.

In line with the previous evidence, the MBCT intervention alleviated the depressive symptoms and increased the mindfulness level. Although the rumination total score did not significantly change after the MBCT in our study, the percentage of reflective rumination increased. Considering that rumination includes depressive, brooding and reflective facets, previous studies noticed that patients with MDD showed more severe rumination with abnormal construct (37, 38), reflecting the interior disturbance in the balance between adaptive and maladaptive rumination. Thus, the relative increase of adaptive, reflective rumination might reflect the process of modulation and rebalance in ruminative responding via the MBCT intervention.

Our results demonstrated increased theta power in the bilateral frontal lobes, the left temporoparietal region and the right occipital region and enhanced phase synchronizations in the theta band mainly in the right hemisphere after MBCT. These results are consistent with previous suggestions that mindfulness meditation produces increased theta power (39, 40), as well as alterations in functional connectivity across brain networks (19, 41, 42). In a pilot study (43), individuals with bipolar disorder showed decreased theta-band power, and post-MBCT individuals showed an increasing trend in theta power. In addition, the regionally increased theta power and phase synchronization after treatment is also in line with previous findings that reported that patients with MDD had less parietal-occipital theta oscillation, abnormal short-range anterior functional connectivity, and asymmetry, as well as a reduced global network strength and clustering coefficient in the theta frequency (23, 24, 44–47). Therefore, our findings indicated that MBCT-induced neural plasticity in theta dynamics reversed, to some extent, abnormalities that occur in MDD.

Further evaluation of the relationship between the MBCT-induced theta alterations and depressive symptoms revealed that the reduced HDRS scores were correlated with theta-band phase synchronizations between the right occipital region and the right prefrontal, central, and parietal regions. Considering the hemispheric asymmetry in emotional processing, our findings in the right hemisphere supported this notion. The oscillatory phase reflects the excitability of neuronal ensembles, and phase synchrony mediates information transfer between oscillating neuronal populations. For example, phase synchrony between anterior and posterior theta rhythms was shown to be involved in successful information encoding (48, 49). Thus, these correlations indicated that the effects on MBCT on the reduction in depressive symptoms were probably mediated by increasing interregional synchrony to regulate emotion-related integration of information in the right hemisphere.

A new and interesting aspect of the present study is that the improvement in reflective rumination exhibited a marked positive correlation with theta power in the left parietal region. There is little direct evidence in the literature regarding MBCT-based neural correlates for alleviating rumination. Based on ongoing experiences, theta oscillations provide an organizing principle of cognitive control, allowing goal-directed behavior. Therefore, the correlates between reflective rumination and theta oscillations appear reasonable could possibly indicate an increased switching between cognitive strategies away from maladaptive and passive processing toward more adaptive, active, and analytical processing.

The topology of the most representative correlations with the changes in reflective rumination after MBCT indicated that the left parietal regions may be critical. Generally, the data presented here are indirectly supported by previous neuroimaging findings. The parietal lobe is known to integrate sensory and egocentric information and be involved in numerical calculations, mental rotation and memory retrieval (50, 51). The left parietal region plays a particularly central role in supporting complex object-directed action, social cognition (e.g., successful perspective-taking and ability to infer thoughts and beliefs), and language processes (52, 53). Parietal regions, such as the inferior parietal lobe (IPL) and precuneus, as the major hub in the DMN, have been verified to, in part, subserve intrinsic awareness and self-referential processing during analytical reflective responses (54–57). Some research has also attempted to regulate functional connectivity between the precuneus and temporoparietal junction to control rumination (57). Therefore, enhanced theta activity in the left parietal region represents the interregional regulation and integration of multidomain informative processing, including concentration and memory retrieval, and switching strategies toward more analytical self-focus after mindfulness training.

Some limitations of the current study must be considered. First, the lack of a control group makes it difficult to reliably interpret the identified oscillation changes and clinical effects as intervention-related findings. Although all the questionnaires were assessed by the certified psychiatrists who did not participant in this intervention study to avoid the subjective expectation, these changes may also reflect longitudinal changes that would have happened without MBCT intervention. By searching for the previous literatures, there were no evidence that the remitted MDD patients with residual symptoms fully recovered without any treatment, and there still were brain abnormalities in the remitted MDD patients as the trait markers of MDD. Although our findings on clinical effects of MBCT and changes in theta oscillation dynamics were consistent with findings reported in previous mindfulness and MDD studies, conclusions from the current design should be interpreted with caution. Second, some findings with statistical significance failed to be corrected considering the multiple comparisons, which may increase the false positive risks. Third, we only focused on theta power and synchronization as the EEG variables of interest in the current pilot study. Given that the roles of different frequency oscillations and cross-frequency interactions in the integration of multiple cognitive functions, other EEG variables and features would probably be affected by the intervention. Thus, more EEG dynamic characteristics which get involved in the mechanisms of MBCT should be explored in the next work. Fourth, considering the low spatial resolution of 18-channel EEG, high-density EEG and combination with MRI would help reveal the neural localization and target of MBCT more precise. Fifth, the lack of a follow-up visit prevents the observation of long-term clinical efficacy and theta alterations. Future studies with larger samples, a control group and longer-term follow-up are needed to further investigate the neural basis of improvements in multidimensional ruminations after mindfulness training.

In conclusion, MBCT alleviated depressive symptoms and improved adaptive reflective rumination in recurrent MDD participants with residual symptoms. By analyzing the power and interregional phase synchronization of EEG oscillations, we observed that the MBCT intervention increased resting-state theta power and reduced theta-band phase synchronization. With regard to the neural correlates, we noticed the strong MBCT effect on theta-band phase synchronizations between the right occipital region and the right prefrontal, central, and parietal regions reduced the depressive symptoms, while theta power in the left parietal region accentuated adaptive, reflective rumination in the recurrent MDD population with residual symptoms. Future studies are warranted to investigate the modulation of theta dynamics in MDD populations with high levels of rumination.

## Data Availability Statement

The raw data supporting the conclusions of this article will be made available by the authors, without undue reservation.

## Ethics Statement

The studies involving human participants were reviewed and approved by the Ethics Committee of the Peking University Shougang Hospital. The patients/participants provided their written informed consent to participate in this study.

## Author Contributions

XY: conceptualization, supervision and project administration. JW: formal analysis, visualization, writing—original draft preparation, and funding acquisition. FR and BG: investigation. FR: resources. FR and XY: writing—review and editing. JW and FR: contributed equally to this work. All authors have read and agreed to the published version of the manuscript.

## Funding

This research was funded by the National Natural Science Foundation of China, Grant Number 81401123.

## Conflict of Interest

The authors declare that the research was conducted in the absence of any commercial or financial relationships that could be construed as a potential conflict of interest.

## Publisher's Note

All claims expressed in this article are solely those of the authors and do not necessarily represent those of their affiliated organizations, or those of the publisher, the editors and the reviewers. Any product that may be evaluated in this article, or claim that may be made by its manufacturer, is not guaranteed or endorsed by the publisher.

## References

[1] GBD 2017 DALYs and HALE Collaborators. Global, regional, and national disability-adjusted life-years (DALYs) for 359 diseases and injuries and healthy life expectancy (HALE) for 195 countries and territories, 1990-2017: a systematic analysis for the Global Burden of Disease Study 2017. Lancet. (2018) 392:1859–922. 10.1016/S0140-6736(18)32335-3 30415748 PMC6252083

[2] Xiao L Feng L Zhu XQ Feng Y Wu WY Ungvari GS. Comparison of residual depressive symptoms and functional impairment between fully and partially remitted patients with major depressive disorder: a multicenter study. Psychiatry Res. (2018) 261:547–53. 10.1016/j.psychres.2018.01.020 29407721

[3] Yang H Chuzi S Sinicropi-Yao L Johnson D Chen Y Clain A. Type of residual symptom and risk of relapse during the continuation/maintenance phase treatment of major depressive disorder with the selective serotonin reuptake inhibitor fluoxetine. Eur Arch Psychiatry Clin Neurosci. (2010) 260:145–50. 10.1007/s00406-009-0031-3 19572158

[4] Nolen-Hoeksema S Wisco BE Lyubomirsky S. Rethinking rumination. Perspect Psychol Sci. (2008) 3:400–24. 10.1111/j.1745-6924.2008.00088.x 26158958

[5] Marchetti I Koster EH Sonuga-Barke EJ De Raedt R. The default mode network and recurrent depression: a neurobiological model of cognitive risk factors. Neuropsychol Rev. (2012) 22:229–51. 10.1007/s11065-012-9199-9 22569771

[6] John E. Roberts EG, Ian H. Gotlib. Ruminative response style and vulnerablility to episodes of dysphoria: Gender, neuroticism, and episode duration. Cogn Therapy Res. (1998) 22:401–23. 10.1023/A:1018713313894

[7] Watkins ER Roberts H. Reflecting on rumination: Consequences, causes, mechanisms and treatment of rumination. Behav Res Ther. (2020) 127:103573. 10.1016/j.brat.2020.103573 32087393

[8] Williams JM Kuyken W. Mindfulness-based cognitive therapy: a promising new approach to preventing depressive relapse. Br J Psychiatry. (2012) 200:359–60. 10.1192/bjp.bp.111.104745 22550328

[9] Cladder-Micus MB Speckens AEM Vrijsen JN AR TD Becker ES Spijker J. Mindfulness-based cognitive therapy for patients with chronic, treatment-resistant depression: a pragmatic randomized controlled trial. Depress Anxiety. (2018) 35:914–24. 10.1002/da.22788 30088834 PMC6175087

[10] Kuyken W Warren FC Taylor RS Whalley B Crane C Bondolfi G. Efficacy of mindfulness-based cognitive therapy in prevention of depressive relapse: an individual patient data meta-analysis from randomized trials. JAMA Psychiatry. (2016) 73:565–74. 10.1001/jamapsychiatry.2016.0076 27119968 PMC6640038

[11] Strauss C Cavanagh K Oliver A Pettman D. Mindfulness-based interventions for people diagnosed with a current episode of an anxiety or depressive disorder: a meta-analysis of randomised controlled trials. PLoS ONE. (2014) 9:e96110. 10.1371/journal.pone.0096110 24763812 PMC3999148

[12] Wang Y Fu C Liu Y Li D Wang C Sun R. A study on the effects of mindfulness-based cognitive therapy and loving-kindness mediation on depression, rumination, mindfulness level and quality of life in depressed patients. Am J Transl Res. (2021) 13:4666–75. 34150046 PMC8205847

[13] Schanche E Vollestad J Visted E Svendsen JL Osnes B Binder PE. The effects of mindfulness-based cognitive therapy on risk and protective factors of depressive relapse - a randomized wait-list controlled trial. BMC Psychol. (2020) 8:57. 10.1186/s40359-020-00417-1 32503649 PMC7275325

[14] Lee DJ Kulubya E Goldin P Goodarzi A Girgis F. Review of the neural oscillations underlying meditation. Front Neurosci. (2018) 12:178. 10.3389/fnins.2018.00178 29662434 PMC5890111

[15] Hudak J Hanley AW Marchand WR Nakamura Y Yabko B Garland EL. Endogenous theta stimulation during meditation predicts reduced opioid dosing following treatment with Mindfulness-Oriented Recovery Enhancement. Neuropsychopharmacology. (2021) 46:836–43. 10.1038/s41386-020-00831-4 32919401 PMC8026958

[16] Schoenberg P Vago DR. Mapping meditative states and stages with electrophysiology: concepts, classifications, and methods. Curr Opin Psychol. (2019) 28:211–7. 10.1016/j.copsyc.2019.01.007 30785068

[17] Tang YY Holzel BK Posner MI. The neuroscience of mindfulness meditation. Nat Rev Neurosci. (2015) 16:213–25. 10.1038/nrn3916 25783612

[18] Cahn BR Polich J. Meditation states and traits: EEG, ERP, and neuroimaging studies. Psychol Bull. (2006) 132:180–211. 10.1037/0033-2909.132.2.180 16536641

[19] Huang FY Hsu AL Chao YP Shang CM Tsai JS Wu CW. Mindfulness-based cognitive therapy on bereavement grief: alterations of resting-state network connectivity associate with changes of anxiety and mindfulness. Hum Brain Mapp. (2021) 42:510–20. 10.1002/hbm.25240 33068043 PMC7775995

[20] Zimmerman B Finnegan M Paul S Schmidt S Tai Y Roth K. Functional brain changes during mindfulness-based cognitive therapy associated with tinnitus severity. Front Neurosci. (2019) 13:747. 10.3389/fnins.2019.00747 31396035 PMC6667657

[21] Lin Y Callahan CP Moser JS. A mind full of self: Self-referential processing as a mechanism underlying the therapeutic effects of mindfulness training on internalizing disorders. Neurosci Biobehav Rev. (2018) 92:172–86. 10.1016/j.neubiorev.2018.06.007 29886175

[22] Qin K Lei D Yang J Li W Tallman MJ Duran LRP. Network-level functional topological changes after mindfulness-based cognitive therapy in mood dysregulated adolescents at familial risk for bipolar disorder: a pilot study. BMC Psychiatry. (2021) 21:213. 10.1186/s12888-021-03211-4 33910549 PMC8080341

[23] Fingelkurts AA Fingelkurts AA. Altered structure of dynamic electroencephalogram oscillatory pattern in major depression. Biol Psychiatry. (2015) 77:1050–60. 10.1016/j.biopsych.2014.12.011 25662102

[24] Shim M Im CH Kim YW Lee SH. Altered cortical functional network in major depressive disorder: a resting-state electroencephalogram study. Neuroimage Clin. (2018) 19:1000–7. 10.1016/j.nicl.2018.06.012 30003037 PMC6039896

[25] Jiang X Shen Y Yao J Zhang L Xu L Feng R. Connectome analysis of functional and structural hemispheric brain networks in major depressive disorder. Transl Psychiatry. (2019) 9:136. 10.1038/s41398-019-0467-9 30979866 PMC6461612

[26] Hamilton JP Farmer M Fogelman P Gotlib IH. Depressive rumination, the default-mode network, and the dark matter of clinical neuroscience. Biol Psychiatry. (2015) 78:224–30. 10.1016/j.biopsych.2015.02.020 25861700 PMC4524294

[27] Jacob Y Morris LS Huang KH Schneider M Rutter S Verma G. Neural correlates of rumination in major depressive disorder: a brain network analysis. Neuroimage Clin. (2020) 25:102142. 10.1016/j.nicl.2019.102142 31901654 PMC6940660

[28] Peters AT Burkhouse KL Kinney KL Phan KL. The roles of early-life adversity and rumination in neural response to emotional faces amongst anxious and depressed adults. Psychol Med. (2019) 49:2267–78. 10.1017/S0033291718003203 30419983 PMC6513724

[29] Chen X Chen NX Shen YQ Li HX Li L Lu B. The subsystem mechanism of default mode network underlying rumination: a reproducible neuroimaging study. Neuroimage. (2020) 221:117185. 10.1016/j.neuroimage.2020.117185 32711069

[30] Hamilton M. A rating scale for depression. J Neurol Neurosurg Psychiatry. (1960) 23:56–62. 10.1136/jnnp.23.1.56 14399272 PMC495331

[31] Han X Yang HF. Chinese version of Nolen-Hoeksema ruminative responses scale (RRS) used in 912 college students: reliability and validity. Chin J Clin Psychol. (2009) 17:549–51.

[32] Wendy Treynor RG Susan Nolen-Hoeksema. Rumination reconsidered: a psychometric analysis. Cogn Therapy Res. (2003). 27:247–59. 10.1023/A:1023910315561

[33] Segal ZV Williams JMG Teasdale JD. Mindfulness Based Cognitive Therapy for Depression. 2nd ed. New York, NY: Guilford Publications (2013).

[34] Wang J Fang Y Wang X Yang H Yu X Wang H. Enhanced gamma activity and cross-frequency interaction of resting-state electroencephalographic oscillations in patients with Alzheimer's disease. Front Aging Neurosci. (2017) 9:243. 10.3389/fnagi.2017.00243 28798683 PMC5526997

[35] Li XL Yao X Fox J Jefferys JG. Interaction dynamics of neuronal oscillations analysed using wavelet transforms. J Neurosci Methods. (2007) 160:178–85. 10.1016/j.jneumeth.2006.08.006 16973218

[36] Lachaux JP Rodriguez E Martinerie J Varela FJ. Measuring phase synchrony in brain signals. Hum Brain Mapp. (1999) 8:194–208. 10.1002/(SICI)1097-0193(1999)8:4<194::AID-HBM4>3.0.CO;2-C 10619414 PMC6873296

[37] Hamilton JP Furman DJ Chang C Thomason ME Dennis E Gotlib IH. Default-mode and task-positive network activity in major depressive disorder: implications for adaptive and maladaptive rumination. Biol Psychiatry. (2011) 70:327–33. 10.1016/j.biopsych.2011.02.003 21459364 PMC3144981

[38] Treynor W Gonzalez R Nolen-Hoeksema S. Rumination reconsidered: a psychometric analysis. Cogn Ther Res. (2003) 27:247–59.

[39] Lomas T Ivtzan I Fu CH. A systematic review of the neurophysiology of mindfulness on EEG oscillations. Neurosci Biobehav Rev. (2015) 57:401–10. 10.1016/j.neubiorev.2015.09.018 26441373

[40] Tang YY Tang R Rothbart MK Posner MI. Frontal theta activity and white matter plasticity following mindfulness meditation. Curr Opin Psychol. (2019) 28:294–7. 10.1016/j.copsyc.2019.04.004 31082635 PMC6778007

[41] Kral TRA Imhoff-Smith T Dean DC Grupe D Adluru N Patsenko E. Mindfulness-based stress reduction-related changes in posterior cingulate resting brain connectivity. Soc Cogn Affect Neurosci. (2019) 14:777–87. 10.1093/scan/nsz050 31269203 PMC6778831

[42] Scult MA Fresco DM Gunning FM Liston C Seeley SH Garcia E. Changes in functional connectivity following treatment with emotion regulation therapy. Front Behav Neurosci. (2019) 13:10. 10.3389/fnbeh.2019.00010 30778290 PMC6369363

[43] Howells FM Ives-Deliperi VL Horn NR Stein DJ. Mindfulness based cognitive therapy improves frontal control in bipolar disorder: a pilot EEG study. BMC Psychiatry. (2012) 12:15. 10.1186/1471-244X-12-15 22375965 PMC3305658

[44] Fingelkurts AA Fingelkurts AA Rytsala H Suominen K Isometsa E Kahkonen S. Impaired functional connectivity at EEG alpha and theta frequency bands in major depression. Hum Brain Mapp. (2007) 28:247–61. 10.1002/hbm.20275 16779797 PMC6871285

[45] Leuchter AF Cook IA Hunter AM Cai C Horvath S. Resting-state quantitative electroencephalography reveals increased neurophysiologic connectivity in depression. PLoS ONE. (2012) 7:e32508. 10.1371/journal.pone.0032508 22384265 PMC3286480

[46] Li Y Kang C Qu X Zhou Y Wang W Hu Y. Depression-related brain connectivity analyzed by EEG event-related phase synchrony measure. Front Hum Neurosci. (2016) 10:477. 10.3389/fnhum.2016.00477 27725797 PMC5035751

[47] Takamiya A Hirano J Yamagata B Takei S Kishimoto T Mimura M. Electroconvulsive therapy modulates resting-state EEG oscillatory pattern and phase synchronization in nodes of the default mode network in patients with depressive disorder. Front Hum Neurosci. (2019) 13:1. 10.3389/fnhum.2019.00001 30774588 PMC6367251

[48] Weiss S Muller HM Rappelsberger P. Theta synchronization predicts efficient memory encoding of concrete and abstract nouns. Neuroreport. (2000) 11:2357–61. 10.1097/00001756-200008030-00005 10943685

[49] Summerfield C Mangels JA. Coherent theta-band EEG activity predicts item-context binding during encoding. Neuroimage. (2005) 24:692–703. 10.1016/j.neuroimage.2004.09.012 15652304

[50] Olson IR Berryhill M. Some surprising findings on the involvement of the parietal lobe in human memory. Neurobiol Learn Mem. (2009) 91:155–65. 10.1016/j.nlm.2008.09.006 18848635 PMC2898273

[51] Wild HM Heckemann RA Studholme C Hammers A. Gyri of the human parietal lobe: volumes, spatial extents, automatic labelling, and probabilistic atlases. PLoS ONE. (2017) 12:e0180866. 10.1371/journal.pone.0180866 28846692 PMC5573296

[52] Garcea FE Mahon BZ. Parcellation of left parietal tool representations by functional connectivity. Neuropsychologia. (2014) 60:131–43. 10.1016/j.neuropsychologia.2014.05.018 24892224 PMC4116796

[53] Bzdok D Hartwigsen G Reid A Laird AR Fox PT Eickhoff SB. Left inferior parietal lobe engagement in social cognition and language. Neurosci Biobehav Rev. (2016) 68:319–34. 10.1016/j.neubiorev.2016.02.024 27241201 PMC5441272

[54] Rosenbaum D Haipt A Fuhr K Haeussinger FB Metzger FG Nuerk HC. Aberrant functional connectivity in depression as an index of state and trait rumination. Sci Rep. (2017) 7:2174. 10.1038/s41598-017-02277-z 28526867 PMC5438394

[55] Berman MG Misic B Buschkuehl M Kross E Deldin PJ Peltier S. Does resting-state connectivity reflect depressive rumination? A tale of two analyses. Neuroimage. (2014) 103:267–79. 10.1016/j.neuroimage.2014.09.027 25264228

[56] Rosenbaum D Int-Veen I Kroczek A Hilsendegen P Velten-Schurian K Bihlmaier I. Amplitude of low frequency fluctuations (ALFF) of spontaneous and induced rumination in major depression: an fNIRS study. Sci Rep. (2020) 10:21520. 10.1038/s41598-020-78317-y 33299001 PMC7725822

[57] Tsuchiyagaito A Misaki M Zoubi OA Tulsa I Paulus M Bodurka J. Prevent breaking bad: A proof of concept study of rebalancing the brain's rumination circuit with real-time fMRI functional connectivity neurofeedback. Hum Brain Mapp. (2021) 42:922–40. 10.1002/hbm.25268 33169903 PMC7856643

